# Antibiotic susceptibility patterns at the Médecins Sans Frontières (MSF) Acute Trauma Hospital in Aden, Yemen: a retrospective study from January 2018 to June 2021

**DOI:** 10.1093/jacamr/dlae024

**Published:** 2024-03-05

**Authors:** Hussein Almehdar, Nagwan Yousef, Wilma van den Boogaard, Amna Haider, Rupa Kanapathipillai, Emad Al-Hodiani, Evgenia Zelikova, Waddah G Moh’d, Justine Michel, Rami Malaeb

**Affiliations:** Médecins Sans Frontières—Operational Centre Paris (MSFOCP), Acute Trauma Hospital, Aden, Yemen; Médecins Sans Frontières—Operational Centre Paris (MSFOCP), Acute Trauma Hospital, Aden, Yemen; Médecins Sans Frontières—Operational Centre Brussels, Medical Department, Luxembourg Operational Research (LuxOR) Unit, Luxembourg City, Luxembourg; Department of Epidemiology and Training, Epicentre, Dubai, United Arab Emirates; Médecins Sans Frontières—Operational Centre Paris, Medical Department, Paris, France; Médecins Sans Frontières—Operational Centre Paris (MSFOCP), Acute Trauma Hospital, Aden, Yemen; Médecins Sans Frontières—Operational Centre Paris, Medical Department, Paris, France; Médecins Sans Frontières—Operational Centre Paris (MSFOCP), Acute Trauma Hospital, Aden, Yemen; Médecins Sans Frontières—Operational Centre Paris, Medical Department, Paris, France; Department of Epidemiology and Training, Epicentre, Dubai, United Arab Emirates

## Abstract

**Background:**

Antimicrobial resistance (AMR) is an urgent global health concern, especially in countries facing instability or conflicts, with compromised healthcare systems. Médecins Sans Frontières (MSF) established an acute trauma hospital in Aden, Yemen, treating mainly war-wounded civilians, and implemented an antimicrobial stewardship (AMS) programme. This study aimed to describe clinical characteristics and identify antibiotic susceptibility patterns representative of patients treated with antibiotics.

**Methods:**

Retrospective cross-sectional study using routinely collected data from all patients treated with antibiotics in the MSF-Aden Acute Trauma hospital between January 2018 and June 2021. Routine clinical data from patients’ files was entered into an AMS electronic database and microbiological data were entered into WHONET. Both databases were imported and merged in REDCap and analysed using RStudio.

**Results:**

Three hundred and sixty-three of 481 (75%) included patients were injured by violence-related trauma. Most were men aged 19–45 years (*n* = 331; 68.8%). In total, 598 infections were diagnosed and treated. MDR organisms were identified in 362 (60.5%) infections in 311 (65%) patients. Skin and soft-tissue infections (SSTIs) (*n* = 143; 24%) were the most common, followed by osteomyelitis (*n* = 125; 21%) and intra-abdominal-infections (IAIs) (*n* = 116; 19%), and 111 (19%) secondary bloodstream infections were identified. *Escherichia coli* was the most frequently identified pathogen, causing IAI (*n* = 87; 28%) and SSTI (*n* = 43; 16%), while *Staphylococcus aureus* caused mainly osteomyelitis (*n* = 84; 19%). Most Gram-negatives were ESBL producers, including *E. coli* (*n* = 193; 81.4%), *Klebsiella pneumoniae* (*n* = 72; 77.4%) and *Enterobacter cloacae* (*n* = 39; 50%) while most *S. aureus* were methicillin resistant (*n* = 93; 72.6%).

**Conclusions:**

High rates of MDR were found. This information will facilitate a comprehensive review of the empirical antibiotic treatment guidelines.

## Introduction

Antimicrobial resistance (AMR) is a global health emergency that is growing at an alarming rate. AMR-related infections are currently responsible for 700 000 deaths annually, and this number is projected to escalate to 10 million deaths in 2050.^1,2^ Low- and middle-income countries (LMICs) carry a disproportionate burden of AMR infections compared with high-income countries. The lack of AMR surveillance and antimicrobial stewardship (AMS), under-resourced laboratories, irrational antibiotic prescription practices and limited infection and prevention control (IPC) measures compound the emergence of AMR and increase the risk of mortality in the population.^3,4^

The emergence and spread of AMR, such as in MRSA, isolates resistant to third-generation cephalosporins, including ESBL-producing pathogens, and other patterns of resistance to the first- and second-line antibiotics in the Middle East region, particularly in conflict-affected areas, is a growing concern.^5^ Such countries are confronted with a high number of war-related injuries that require advanced medical care, strict IPC measures and necessitate appropriate and curative antibiotic therapy to manage the risk of AMR infections.^6^ Despite the limited number of studies conducted in conflict-affected countries, the available evidence suggests a high prevalence of AMR. Studies from Iraq, Syria and Yemen have reported high levels of AMR among commonly used antibiotics.^5–7^ One study focused on Yemeni children aged 1 to 15 years treated for otitis media, and another study examined adults in Aden; both showed widespread resistance to commonly used antibiotics.^8,9^ Another study conducted among adults in Sana’a reported high resistance patterns for common bacterial isolates in ophthalmic patients.^10^ High rates of antibiotic misuse are reported in Yemen, with self-medication estimated to be as high as 78%.^11^ This may be a contributing factor to the observed resistance, which is among the highest in the region.^7,9^ Furthermore, a systematic review on AMR in Yemen highlighted the lack of reliable data and the urgent need for increased surveillance and monitoring of AMR.^5^

Médecins Sans Frontières (MSF), an international, independent medical humanitarian organization, established a trauma hospital in Aden in 2012, which treats war-related injuries for both soldiers and civilians. In 2017, an AMS programme was introduced to ensure proper antibiotic use and safety. A national Yemeni doctor received appropriate training to implement the programme based on MSF protocols and international standards then received frequent coaching and support from infectious disease referents regionally and in the headquarters. MSF-Aden now has an effective programme in place, including daily feedback on laboratory results and regular audits of prescribing practices. The hospital introduced an empirical treatment guideline in 2018, but it has not been updated since. This study aims to describe the clinical characteristics of patients and their infection diagnoses treated with antibiotics between January 2018 and June 2021 to update the empirical antibiotic treatment guidelines with up-to-date local antibiogram data.

## Materials and methods

### Settings

#### The MSF Aden Acute Trauma Hospital

MSFs Aden hospital, opened in 2012, treats war-related injuries regardless of whether the patient is a soldier or civilian. It has an emergency room, an ICU and an outpatient department, with a total of 81 beds. A microbiology laboratory and two isolation wards for MDR infections were added in 2017. Over 50 000 patients have been treated. An AMS programme and IPC measures were implemented to ensure proper antibiotic use and safety.

#### Study design and population

This is a retrospective cross-sectional study using routinely collected data. The study included all patients who were admitted and treated with antibiotics for different types of infections at the MSF Aden Acute Trauma hospital between January 2018 and June 2021.

#### Identification of bacterial isolates and antibiotic susceptibility testing (AST)

Samples of blood and urine were taken from patients who showed signs of infection as part of a sepsis screening protocol. Blood cultures were taken routinely for patients with severe infections, including intra-abdominal infections, pneumonia, necrotizing fasciitis, line infections, septic shock and unexplained continuous fever. Urine cultures were collected from patients with symptoms of urinary tract infections (UTIs). In cases where the source of infection was unknown, both blood and urine cultures were taken. Intraoperative samples were also taken from infected wounds after washout and debridement procedures. All sample collections were performed with strict sterile techniques in accordance with MSF microbiology sample collection standard operating procedures (SOPs). A clinical microbiology laboratory technician performed culture and AST. Main culture media were blood CNA (colistin and nalidixic acid), chocolate PVX (PolyViteX supplement) and MacConkey and CHROMagars. Species were identified through manual flowcharts (API Gallery System).

Antibiotic susceptibilities were determined by manual disc diffusion (Bio-Rad and Oxoid), concentration gradient test and broth microdilution test (Liofilchem). They were interpreted using the EUCAST guideline.^12^ An isolate was defined as MDR if it showed non-susceptibility to at least one agent in ≥3 antimicrobial categories. The diagnosis of wound infection was based on clinical signs of infection with or without confirmatory positive microbiological culture.

#### Data collection and analysis

The routinely gathered hospital data, including clinical and demographic characteristics, were collected by the dedicated AMS doctor and entered into a REDCap 12.2.0 software dedicated study database. Culture and sensitivity results were obtained from the WHONET database;^13^ WHONET data for the patients who were not treated by antibiotics were excluded from this study analysis. Descriptive statistics were used to characterize patients’ demographic and clinical data. Pathogen identification and antibiotic susceptibility were described based on the EUCAST guidelines.^12^ Stratified analysis was used to assess the difference in outcomes, and Fisher’s exact tests were used for categorical variables and parametric or non-parametric tests were used for numerical variables. The data were analysed using RStudio v.1.2.5.033 statistical software.

## Ethics

The study protocol was approved by the Ethical Research Committee of the Faculty of Medicine and Health Sciences in Aden, Yemen (Research code: REC-106-2021) and was exempted by the MSF-OCP medical director, mandated by the MSF Ethics Review Board.

## Results

### Baseline characteristics and prevalence of MDR infections

The study included a total of 481 patients. Most patients were male (88%) and in the 19–45 age group (68.8%). Violence-related trauma was the leading cause of admission (75%), mainly due to gunshots (64%). Penetrating wounds were the most frequent nature of trauma (46%). The abdomen (47%) and lower limb (44%) were the most commonly affected injury sites. Three hundred and-eleven (65%) patients were diagnosed with at least one MDR infection, while the remaining 170 (35%) had non-MDR infections, including those with no conclusive bacterial growth results or were not sampled (Table 1).

**Table 1.** Demographic and clinical characteristics of antibiotic-treated patients by MDR status at the MSF Aden Acute Trauma Hospital, Yemen, January 2018—June 2021

Characteristic	Total *N* = 481	Non-MDR-infected patients, *N* = 170 (35%)^a^	MDR-infected patients *N* = 311 (65%)	P value
Sex, *n* (%)				
Male	423 (88)	147 (86)	276 (89)	0.5
Female	58 (12)	23 (14)	35 (11)	
Age group, years, *n* (%)				
1–18	100 (21)	38 (22)	62 (20)	0.2
19–25	161 (33.5)	64 (38)	97 (31)	
26–45	170 (35.3)	57 (33)	113 (36)	
46–65	44 (9)	10 (6)	34 (11)	
66–95	6 (1.2)	1 (1)	5 (2)	
Year of injury, *n* (%)				
2018	148 (31)	51 (30)	97 (31)	0.2
2019	141 (29)	44 (26)	97 (31)	
2020	143 (30)	51 (30)	92 (30)	
2021	49 (10)	24 (14)	25 (8)	
Violence-related trauma, *n* (%)	363 (75)	109 (64)	254 (82)	<0.001
Cause of admission, *n* (%)				
Gunshot	306 (64)	95 (56)	211 (68)	0.003
Road traffic accident	79 (16)	41 (24)	38 (12)	<0.001
Bomb/mine explosion	59 (12)	12 (7)	47 (15)	0.012
Infection	13 (3)	11 (6.5)	2 (1)	<0.001
Other	24 (5)	11 (6.5)	13 (4)	0.3
Number of injury sites, *n* (%)				
One	333 (69)	118 (69)	215 (69)	0.9
Two	87 (18)	32 (19)	55 (18)	
Three or more	61 (13)	20 (12)	41 (13)	
Injury site, *n* (%)				
Abdomen	228 (47)	71 (42)	157 (50)	0.067
Lower limb	213 (44)	75 (44)	138 (44)	>0.9
Thorax	77 (16)	36 (21)	41 (13)	0.022
Upper limb	51 (11)	23 (14)	28 (9)	0.12
Pelvis	49 (10)	16 (9.4)	33 (11)	0.7
Head/neck	13 (2.7)	4 (2.4)	9 (2.9)	>0.9
Nature of trauma, *n* (%)				
Penetrating wound	221 (46)	64 (38)	157 (50)	0.007
Open fracture	190 (40)	64 (38)	126 (41)	0.5
Soft tissue	68 (14)	27 (16)	41 (13)	0.4
Blunt	31 (6.4)	16 (9.4)	15 (4.8)	0.05
Closed fracture	27 (5.6)	14 (8.3)	13 (4.2)	0.065
Traumatic amputation	23 (4.8)	4 (2.4)	19 (6.1)	0.065
Vascular	21 (4.4)	5 (2.9)	16 (5.1)	0.3
Number of diagnosed infections, *n* (%)				
One	390 (81)	151 (89)	239 (77)	0.005
Two	72 (15)	16 (9)	56 (18)	
Three or more	19 (4.0)	3 (2)	16 (5)	

Numbers of injury sites and nature of trauma add up to more than 481 because of polytraumatic injuries that can involve more than one site and nature of injure in one patient.

^a^Fifty-seven patients had no growth from their samples, or no samples were taken.

Patients with violence-related trauma had a significantly higher proportion of MDR infections (82%) compared with those without violence-related trauma (64%) (*P* < 0.001). The proportion of MDR infections was significantly higher in patients with gunshot injuries (68%) compared with those with non-gunshot injuries (32%) (*P* = 0.003). Patients with MDR infections were more likely to have multiple infections diagnosed (23% versus 11%) (*P* = 0.005). In addition, patients with penetrating wounds and abdominal injuries were more likely to have MDR infections compared with other types of trauma and injury sites (*P* = 0.007 and *P* = 0.067, respectively) (Table 1).

### Infection diagnoses and isolated bacteria

**Figure 1.**
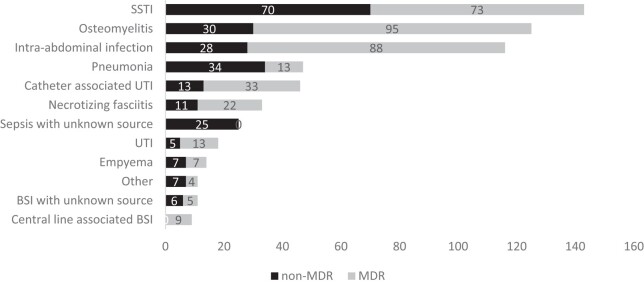
Frequency of 598 infections by diagnosis and MDR status at the MSF Aden Acute Trauma Hospital, Yemen, January 2018—June 2021.

The analysis of the 598 infections revealed that skin and soft-tissue infection (SSTI) (*n* = 143; 24%) was the most common infection diagnosis, followed by osteomyelitis (*n* = 125; 21%) and intra-abdominal infection (IAI) (*n* = 116; 19%). MDR infections (*n* = 362; 60.5%) were more common than non-MDR infections (*n* = 236; 39.5%) in most infection diagnoses, except for blood stream infection (BSI) with unknown source (*n* = 5; 45%) and pneumonia (*n* = 13; 28%) (Figure 1).

**Figure 2.**
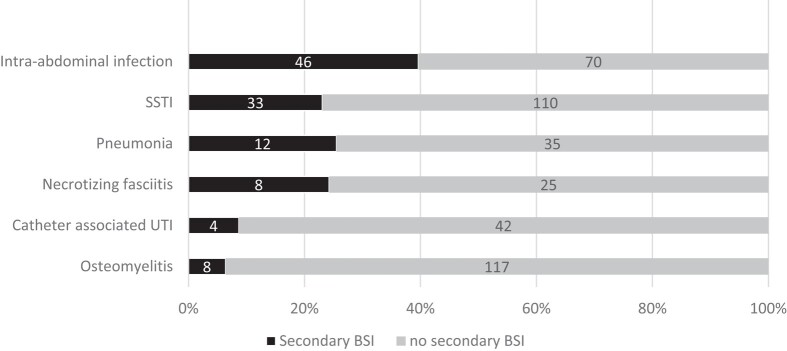
Incidence of 111 BSIs by infection diagnosis at the MSF Aden Acute Trauma Hospital, Yemen, January 2018—June 2021.

From the same 598 infections, 111 (19%) were also diagnosed with secondary BSI, (*n* = 60; 54%) confirmed through matched positive growth in the peripheral blood sample and primary source samples, while others (*n* = 51; 46%) had positive peripheral blood samples only with clinically diagnosed and a highly suspected primary source. The most common infections associated with secondary BSI were IAI (*n* = 46; 41%) and SSTI (*n* = 33; 30%). In contrast, osteomyelitis and catheter-associated UTI (CAUTI) had lower incidence of secondary BSI (≤8 cases) (Figure 2).

**Figure 3.**
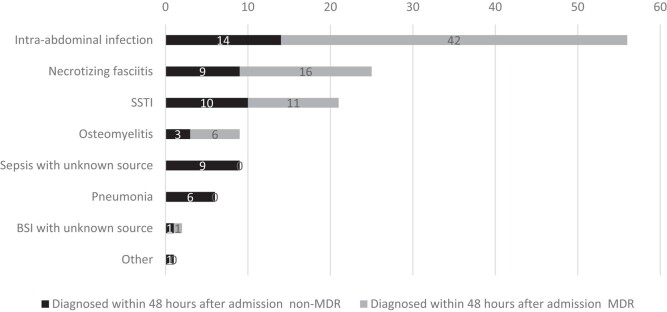
Frequency of the types of the 129 infections diagnosed within 48 h of admission by MDR status at the MSF Aden Acute Trauma Hospital, Yemen, January 2018—June 2021.

**Figure 4.**
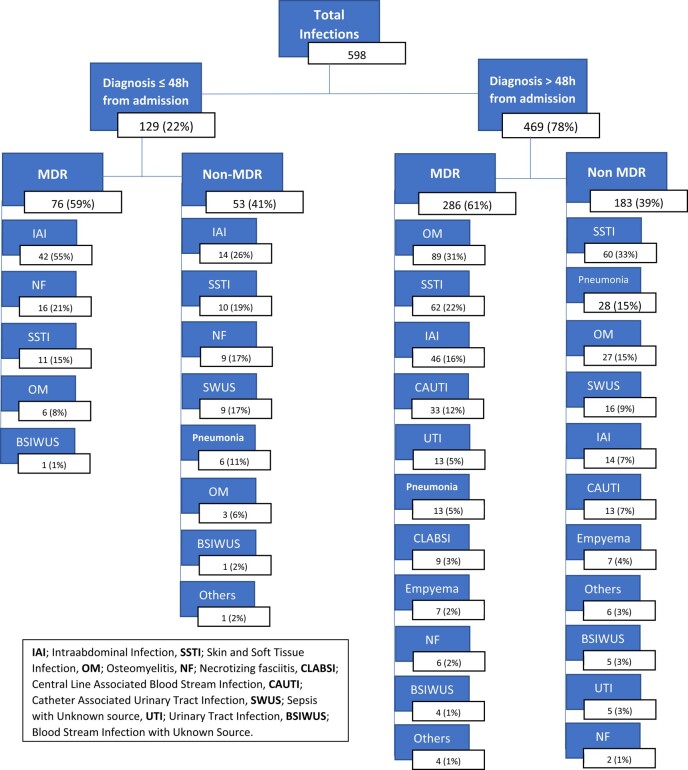
Distribution of the 598 infections diagnosed according to time of diagnosis and MDR status at the MSF Aden Acute Trauma Hospital, Yemen, January 2018—June 2021.

Overall, 129 (22%) infections were diagnosed within 48 h of admission; 59% of these were caused by MDR organisms. The most common diagnoses were IAI (*n* = 56; 43%), followed by necrotizing fasciitis (*n* = 25; 19%) and SSTI (*n* = 21; 16%) (Figures 3 and 4).

Of the 598 infection diagnoses, 1249 bacterial isolates comprised 52 different species. The most common isolates were *Escherichia coli* (*n* = 238; 19%), followed by *Enterococcus faecalis* (*n* = 154; 12%) and *Staphylococcus aureus* (*n* = 129; 10%). The distribution of bacterial species responsible for these infections varied widely among different infection diagnoses (Table 2). For example, *E. coli* was the most common bacterial species responsible for causing IAI (*n* = 87; 28%), SSTI (*n* = 43; 16%), necrotizing fasciitis (*n* = 18; 26%), CAUTI (*n* = 29; 44%) and UTI (*n* = 9; 53%), while *E. faecalis* was mostly isolated from patients diagnosed with IAI (*n* = 52; 17%) and osteomyelitis (*n* = 50; 12%). *S. aureus* was mainly isolated from those with osteomyelitis (*n* = 84; 19%).

**Table 2.** Distribution of 1249 bacterial isolates by infection diagnoses at the MSF Aden Acute Trauma Hospital, Yemen, January 2018—June 2021

	Osteomyelitis (N = 432)	IAI (N = 312)	SSTI (N = 263)	Necrotic zing fasciitis (N = 70)	CAUTI (N = 66)	Pneumonia (N = 30)	Empyema (N = 23)	UTI (N = 17)	BSI with unknown source (N = 13)	Central line-associated BSI (N = 10)	Other (N = 13)
*E. coli*, *n* (%)	41 (10)	87 (28)	43 (16)	18 (26)	29 (44)	2 (7)	3 (13)	9 (53)	3 (23)	1(10)	2 (15)
*E. faecalis*, *n* (%)	50 (12)	52 (17)	28 (11)	13 (19)	2 (3)	—	3 (13)	—	3 (23)	—	3 (23)
*S. aureus*, *n* (%)	84 (19)	4 (1)	28 (11)	1 (1.5)	3 (4)	1 (3)	2 (9)	—	1 (8)	3(30)	2 (15)
*P. aeruginosa*, *n* (%)	44 (10)	17 (5)	31 (12)	3 (4.5)	6 (9)	4 (13)	3 (13)	—	—	—	1 (8)
*K. pneumoniae*, *n* (%)	24 (6)	28 (9)	17 (6)	10 (14)	5 (8)	2 (7)	3 (13)	2 (12)	1 (8)	—	1 (8)
*P. mirabilis*, *n* (%)	27 (6)	27 (9)	14 (5)	4 (6)	7 (11)	3 (10)	3 (13)	2 (12)	—	—	—
*A. baumannii*, *n* (%)	9 (2)	25 (8)	27 (10)	4 (6)	1 (1.5)	11 (37)	2 (9)	—	3 (23)	5(50)	—
*E. cloacae*, *n* (%)	31 (7)	11 (3)	21 (8)	8 (11)	4 (6)	—	—	2 (12)	—	1(10)	2 (15)
*Streptococcus pyogenes*, *n* (%)	24 (6)	—	7 (3)	1 (1)	—	1 (3)	—	—	—	—	—
*E. faecium*, *n* (%)	3 (1)	15 (5)	7 (3)	3 (4)	—	—	—	1 (6)	1 (8)	—	—
CoNS*, n* (%)	11 (2)	9 (3)	4 (1)	—	1 (1.5)	1 (3)	2 (9)	1 (6)	—	—	1 (8)
Other, *n* (%)	84 (19)	37 (12)	36 (14)	5 (7)	8 (12)	5 (17)	2 (9)	—	1 (8)	—	1 (8)

Percentages in columns may not sum to exactly 100% due to rounding.

### Antibiotic susceptibility and resistance mechanisms

Ampicillin showed low effectiveness against Enterobacterales, with only 3.4% of *E. coli*, 1.3% of *Enterobacter cloacae* and 1.1% of *Klebsiella pneumoniae* being susceptible. Ceftriaxone also showed low effectiveness, with susceptibility rates of only 14.8% for *E. coli* and 19.4% for *K. pneumoniae*. Piperacillin/tazobactam, ertapenem and amikacin were the most effective antibiotics, with high percentages of susceptible isolates, ranging from 67.7% to 100% (Table 3).

**Table 3.** Antibiotic susceptibility of the most common isolates from different sites (blood, urine, bone, tissue and fluid aspirates) and causing different types of infections in the patients treated with antibiotics at the MSF Aden Acute Trauma Hospital, Aden, Yemen, January 2018–June 2021

	No. of isolates tested (% susceptible)^a^																				
	Ampicillin	Amoxicillin/clavulanic acid	Piperacillin/tazobactam	Cefoxitin	Ceftazidime	Ceftriaxone	Cefepime	Aztreonam	Ertapenem	Imipenem	Amikacin	Gentamicin	Tobramycin	Ciprofloxacin	Levofloxacin	Trimethoprim/sulfamethoxazole	Clindamycin	Rifampicin	Vancomycin	Tigecycline	Colistin
Enterobacterales																					
*E. coli*	237 (3.4)	237 (57)	237 (78.5)	237 (84.4)	235 (15.7)	237 (14.8)	237 (15.6)	237 (14.8)	226 (93.4)	—	237 (97)	237 (51.5)	237 (45.1)	237 (37.1)	44 (34.1)	237 (36.7)	—	—	—	—	—
*K. pneumoniae*	93 (1.1)	93 (55.9)	93 (67.7)	93 (92.5)	93 (29)	93 (19.4)	93 (20.4)	93 (23.9)	93 (96.8)	—	93 (98.9)	93 (44.1)	93 (37.6)	93 (36.6)	—	93 (31.2)	—	—	—	—	—
*E. cloacae*	77 (1.3)	78 (16.7)	77 (74)	78 (17.9)	77 (42.9)	78 (44.9)	78 (47.4)	78 (43.6)	69 (98.6)	—	78 (98.7)	78 (46.2)	77 (48.1)	78 (50)	—	78 (53.8)	—	—	—	—	—
*P. mirabilis*	86 (48.8)	86 (94.2)	86 (97.7)	86 (98.8)	86 (72.1)	86 (66.3)	86 (65.1)	86 (72.1)	82 (100)		86 (75.6)	86 (70.9)	86 (67.4)	86 (68.6)	—	86 (55.8)	—	—	—	—	—
^b^Gram-negative																					
*P. aeruginosa*	—	—	106 (86.8)	—	106 (83)	—	106 (85.8)	106 (0.9)	—	106 (92.5)	106 (89.6)	106 (84)	106 (84)	106 (91.5)	—	—	—	—	—	—	—
*A. baumannii*	—	—	—	—	—	—	—	—	—	87 (3.5)	87 (39.1)	87 (2.3)	84 (4.8)	87 (12.6)	—	—	—	—	—	—	49 (100)
Gram-positive																					
*S. aureus*	—	—	—	128 (27.4)	—	—	—	—	—	—	128 (97.7)	128 (93.8)	128 (93)	128 (85.9)	—	128 (99.2)	128 (85.9)	127 (97.6)	90 (100)	—	—
*E. faecalis*	151 (94)	—	—	—	—	—	—	—	—	—	—	—	—	—	—	—	—	—	151 (100)	—	—
*E. faecium*	30 (13.3)	—	—	—	—	—	—	—	—	—	—	—	—	—	—	—	—	—	30 (100)	—	—
*Streptococcus pyogenes*	—	—	—	—	—	—	—	—	—	—	—	—	—	—	—	—	32 (100)	—	—	—	—

^a^AST results are only displayed for results found for 30 isolates or more of the same bacterial species.

^b^Non-fermenting Gram-negative bacteria.

*Pseudomonas aeruginosa* had low susceptibility to aztreonam, with only 0.9% of isolates being susceptible. Other antibiotics such as piperacillin/tazobactam, ceftazidime, cefepime and imipenem showed high effectiveness against this bacterial species, with susceptible isolates ranging from 83% to 92.5%. Moreover, imipenem, gentamicin, tobramycin and ciprofloxacin showed low effectiveness against *Acinetobacter baumannii* isolates, susceptibility ranging from 2.3% to 12.6%, while it was 100% susceptible to colistin. *S. aureus* showed high susceptibility to most of the tested antibiotics, except for cefoxitin (27.3%). *E. faecalis* and *Enterococcus faecium* showed 100% susceptibility to vancomycin, but ampicillin was more effective against *E. faecalis* (94%) compared with *E. faecium* (13.3%).

Table 4 shows that *S. aureus* had a high rate of resistance to methicillin (72.6% of isolates). *E. coli* had the highest percentage of ESBL-producing isolates, at 81.4%, followed by *K. pneumoniae* with 77.4% and *E. cloacae* with 50%. *A. baumannii* showed the highest percentage of carbapenem-resistant isolates at 96.5%. *P. aeruginosa* had a low percentage of carbapenem-resistant isolates, at 7.5%; however, it had a 15% rate of ceftazidime-resistant isolates.

**Table 4.** Resistance patterns of the most commonly isolated bacteria from patients treated with antibiotics at the MSF Aden Acute Trauma Hospital, Yemen, January 2018–June 2021

Pathogen	Total (*n*)	MRSA, *n* (%)	ESBL, *n* (%)	Carbapenem resistant, *n* (%)	PARC, *n* (%)
*S. aureus*	128	93 (72.6)	__	__	__
*E. coli*	237	__	193 (81.4)	6 (2.5)	__
*K. pneumoniae*	93	__	72 (77.4)	3 (3.1)	__
*E. cloacae*	78	__	39 (50)	2 (2.5)	__
*P. mirabilis*	86	__	29 (33.7)	__	__
*A. baumannii*	87	__	__	84 (96.5)	__
*P. aeruginosa*	106	__	__	8 (7.5)	16 (15)

PARC, *P. aeruginosa* resistant to ceftazidime.

## Discussion

To our knowledge, this study is the first to provide a detailed description of infections and antimicrobial resistance patterns among trauma patients in Yemen. The study found a high prevalence of MDR infections among patients treated with antibiotics for conflict-related injuries, with 65% of patients affected. The most common infections were SSTIs, followed by osteomyelitis, IAIs and BSIs. MDR organisms were identified in 60.5% of infections, while violence-related trauma, particularly gunshot injuries, were strongly associated with MDR infections. Patients with MDR infections were also more likely to have multiple infection diagnoses.

During the study period, Yemen was experiencing intense fighting, which led to a significant rise in casualties and civilian injuries, primarily caused by shootings, bombings and other armed violence against civilians.^14^ Consequently, most trauma patients treated at the MSF Aden Acute Trauma Hospital were victims of war-related injuries, mainly affecting the young male population. The mechanism and types of injuries observed, predominantly characterized by gunshots and penetrating wounds, were similar to other conflict settings reported in the Middle East, including Syria, Iraq and Lebanon.^15–17^ Recent armed conflicts are marked by higher usage of warfare technology that correlate with more severe war injuries. This also leads to a high risk of mortality, and complications, as well as increased costs of treatment.^18^ Injuries sustained during armed conflicts are frequently complex, with a high risk of contamination due to foreign objects. This often leads to severe complications, resulting in a significant number of tissue infections, bone infections and BSIs,^19^ as was also observed in our study.

Secondary BSI represented 19% of all treated infections and the main source of secondary BSI (41%) was IAI in our trauma centre. Interestingly, this was different from a prospective study done in a level one trauma centre in Jai Prakash Narayan Apex Trauma Centre (JPNATC), India, where ventilator-associated pneumonia was the most common source.^20^ As we found in our study, IAI and BSI were also among the three infectious diagnoses dominating the global burdens associated with AMR in a global systemic analysis done in 2019.^21^

In our study, we found that *E. coli* was the most frequently isolated bacterium (19%) among patients treated for underlying infections, followed by *E. faecalis* (12%), *S. aureus* (10%), *P. aeruginosa* (8.7%) and *K. pneumoniae* (7.4%). We were unable to compare our results with previous studies of trauma patients only in Yemen, but a recent study conducted in Aden including a mix of patients from multiple hospitals and medical laboratories showed different findings, as here *Staphylococcus* spp. was the most commonly isolated bacteria (41.7%), followed by *E. coli* (39.8%), *Pseudomonas* spp. (8.9%) and *K. pneumoniae* (4.36%).^9^ The difference in the most commonly found organisms between our study and the previously mentioned study could be due to the type of patients, injuries, diagnoses and samples studied. The other study included multiple centres, mixed patients with wounds, and pus superficial swabs, while our study focused on conflict-related traumatic injuries and analysed deep tissue and bone samples in one centre. These factors, particularly sample type, may have influenced the prevalence of certain organisms in each study.^22^ Another study among Yemeni patients treated for osteomyelitis in an MSF reconstructive surgery hospital in Amman, Jordan showed that infections were mainly caused by *S. aureus.*^23^ Other comparisons with conflict settings in the Middle East showed some similarities with our findings, although the prevalence of the isolated bacteria was highly influenced by the study methodologies, sample size and study population.^24–26^

In Palestine, among patients with postoperative surgical site infections (SSIs), findings showed prevalence of *E. coli* (56.7%), *S. aureus (30%)*, *Klebsiella* spp. (6.7%) and *A. baumannii* (3.3%),^24^ and another study from Iraq but with a smaller sample size of patients (*n* = 174) compared with our study, showed that *S. aureus* (48.2%) was the most common isolated bacterium, followed by Enterobacterales including *Proteus mirabilis*, *E.coli*, *E. cloacae* and *K. pneumoniae* and *P. aeruginosa* together representing 35.9%.^25^ Moreover, in a surgery hospital in Lebanon, treating acute and chronic war-related trauma patients, predominantly from the Syrian war, where *S. aureus* (49.1%) was the most commonly isolated bacterium, followed by Enterobacterales (28.5%) and *P. aeruginosa* (13.2%) but only from bone and tissue samples.^26^

MRSA infections are a public health concern as hospital-acquired MRSA infection rates have slowly increased over the last 25 years and find their way into the community.^27^ However, other studies show that community-acquired MRSA and hospital-acquired MRSA possess different and specific virulence factors and toxins.^28^

The prevalence of MRSA across conflict regions is generally high,^29^ with a range of MRSA from all *S. aureus* isolates of 72.6% in our hospital to 95.4% in Mosel, Iraq^25^ and 48.5% in one of Lebanon’s studies.^26^

As observed in our study, *S. aureus* and particularly MRSA was mainly identified from bone samples as a major pathogen for osteomyelitis in the trauma centre in Lebanon.^26^

ESBL-producing organisms are another emerging challenge, as they causes nosocomial and community-acquired infections.^30^ In our study, we observed a high rate of ESBL-producing Enterobacterales, with *E. coli* (81.4%), *K. pneumoniae* (77.4%) and *E. cloacae* (50%) being the most common. This was also seen in the Mosel study,^25^ where even higher rates of ESBL-producing Enterobacterales were reported in Jordan with *E. cloacae* (88.2%), whereas, ESBL production in *E. coli* was much lower (10.8%).^31^ Iran showed similarities concerning *E. coli* (89.8%) and *K. pneumoniae* (72.1%) but had other results with *A. baumannii* (84.2%) and *P. aeruginosa* (83.8%).^32^ Carbapenems are usually recognized as the drug of choice to treat severe infections caused by ESBL-producing pathogens.^30^ However, their overuse has already led to the emergence of resistance, necessitating the exploration of carbapenem-sparing strategies and effective agents against MDR pathogens.^30^ Piperacillin/tazobactam and cefoxitin have been reviewed as potential carbapenem-sparing agents for mild to moderate infections caused by ESBL-producing pathogens,^33,34^ but their efficacy varies in severe infections such as IAIs, bone infections and BSIs. Here, the optimal efficacy of its usage is still unclear and may be dependent on *in vitro* susceptibility, MICs and/or the site and severity of the infection.^33–36^ In our study, some ESBL-producing Enterobacterales showed a relatively high-rate prevalence of susceptibility to them *in vitro*, although their potential use in our hospital might be limited due to the lack of an MIC-reporting method in our laboratory, and the site or severity and/or complications of the infections we deal with in our acute surgical trauma setting.^33–36^

Fluoroquinolones and trimethoprim/sulfamethoxazole are other carbapenem-sparing and de-escalating agents that could be the best choice for an IV-to-oral switch therapy for infections caused by ESBL-producing pathogens.^35^ However, in our study, these MDR infections showed low susceptibility to treatment with these agents, leading to longer hospitalization stays and more pressure on carbapenem use, especially for infections that required long-term antibiotic therapy, such as bone infections. The most common carbapenem-resistant Enterobacterales showed low reported prevalence rates (2.5%–3.1%) in our study, similar to findings in Haiti (2.6%).^29^ However, carbapenem resistance in *P. aeruginosa* showed a totally different prevalence, with high rates in Haiti (26.9%), Palestine (47.6%), lower rates in Iraq (12.4%), and much lower rates in our hospital (7.5%). These variations in *P. aeruginosa* findings might need further in-depth analysis to explore relevant correlated and risk factors.

MDR *A. baumannii* has been globally recognized as an emerging nosocomial pathogen.^37–39^ It has been identified with high rates among war-wounded patients in different conflict zones, including the Middle East region^40^ Carbapenem-resistant *A. baumannii* was found at a very high rate (96.5%) in our study, which is very similar to other studies from conflict-affected settings in the region (78%), but also in non-conflict settings (69%–75%).^5^ Despite high susceptibility (100%) of MDR *A. baumannii* to colistin in our study, colistin is a drug with an unfavourable side-effect profile, and pharmacokinetics that limit its effect in some sites of infection.^41,42^ Therefore, creating access to safe and efficacious new therapeutics, like novel new-generation cephalosporins, is an urgent need in contexts with high rates of MDR *A. baumannii* infections.^43^

Lastly, in a 2019 systemic analysis of the AMR global burden, *E. coli*, *S. aureus*, *K. pneumoniae* and *A. baumannii* were among six leading pathogens responsible for more than 250 000 deaths attributed to AMR; this may support the results of high-prevalence AMR patterns seen among these pathogens in this study, as shown in Table 4.^21^

AMR is a significant public health challenge in Yemen,^44^ although its actual extent is unknown. Available data are very limited due to the lack of access to equipped microbiological laboratories to monitor the patterns of AMR across the country.^45^ In 2018, the MSF hospital adapted its empirical antibiotic treatment based on a 1 year review of the microbiology hospital data. As a result of this review, many of the usual first-line antibiotics were replaced by second- and third-line antibiotics considering the high level of AMR seen among the patients treated that year. For instance, the guide suggested vancomycin and carbapenem as the first choice for sepsis/septic shock syndrome empirical treatment after taking blood and other possible samples and pending microbiology results. Our practice is guided by the principle of ‘Start smart, then focus’. This means that when sepsis is suspected, we endeavour to identify and sample the likely source, initiating empirical treatment based on the recommended guidelines for that suspected source. In instances where the source of sepsis is not immediately apparent, a diagnosis of sepsis with an unknown source is made, pending results from a comprehensive septic screen targeting all possible and accessible sources (such as blood, lines, urine, lungs, etc.). For these patients, vancomycin and meropenem may be started empirically. However, these treatments are tailored as soon as possible, based on microbiology laboratory results and/or the clinical evaluation of the patient, which may reveal a more obvious source of infection.

Concerning nurturing an evaluation of the empirical antibiotic guideline, our study findings do support the existing MSF empirical treatment guidelines that were implemented in 2018 and confirm the need for second-line antibiotics such as vancomycin and a carbapenem for empirical therapy of sepsis and septic shock from most common infections seen, like IAI and SSTI.

Ensuring access to reliable microbiology, these and other affordable quality-assured second-line antibiotics, and anticipating the need for, and ensuring affordable access to third-line antibiotics as rates of carbapenemases increase, is essential. Rigorous IPC efforts and AMS must be expanded to address these high rates of MDR.

This study has limitations that should be noted. Firstly, it was conducted in one hospital in Aden and only included trauma patients who met specific admission criteria. This means that the findings may not be generalizable to other trauma patients in Yemen, and certain traumas, such as head injuries, were excluded. Additionally, the study relied on routinely collected data, which may not be completely reliable or available. However, missing or erroneous data were checked and corrected by the principal investigators. Another limitation is that the microbiology laboratory was unable to confirm carbapenem mechanisms of resistance, which would have provided valuable insights into the risk of AMR. Despite these limitations, the study had strengths, such as a large sample size over multiple years and high-quality laboratory data. The study also provided insights into the AMR patterns underlying the most common infectious complications in trauma patients in Yemen.

### Conclusions

The prevalence of AMR among patients with acute trauma injuries in Aden, Yemen is alarmingly high and underscores the urgent need for context-specific antibiotic treatment guidelines, applicable IPC strategies, access to reliable microbiological diagnostics, and clinical and microbiological surveillance. Overall, the study highlights the need for a collaborative effort to improve AMS, IPC preventive measures, and source control by appropriate surgical management, in order to combat the spread of MDR infections in conflict-affected areas.
